# Initial In Vivo Evaluation of a Novel Amikacin-Deoxycholate Hydrophobic Salt Delivers New Insights on Amikacin Partition in Blood and Tissues

**DOI:** 10.3390/pharmaceutics13010085

**Published:** 2021-01-10

**Authors:** Styliani Xiroudaki, Federica Ianni, Samuele Sabbatini, Elena Roselletti, Claudia Monari, Roccaldo Sardella, Anna Vecchiarelli, Stefano Giovagnoli

**Affiliations:** 1 Department of Pharmaceutical Sciences, Via del Liceo 1, 06123 Perugia, Italy; styliani.xiroudaki@studenti.unipg.it (S.X.); federica.ianni@unipg.it (F.I.); roccaldo.sardella@unipg.it (R.S.); 2 Department of Medicine, Medical Microbiology Section, University of Perugia, Piazzale Gambuli 1, 06132 Perugia, Italy; samuele.sabbatini@gmail.com (S.S.); elenaro@hotmail.it (E.R.); claudia.monari@unipg.it (C.M.); anna.vecchiarelli@unipg.it (A.V.)

**Keywords:** amikacin, amikacin-deoxycholate hydrophobic salt, pulmonary delivery, pharmacokinetics, inflammation, amikacin partition

## Abstract

In this study, an initial in vivo evaluation of a new amikacin-deoxycholate hydrophobic salt aimed at potentiating amikacin action against hard-to-treat lung infections was undertaken by quantifying, for the first time, amikacin in whole blood. Pharmacokinetic evaluation after intranasal administration in a murine model showed higher drug retention in the lungs compared to blood, with no significant differences between the salt and the free drug. Upon repeated administrations, the two treatments resulted in nonsignificant tissue damage and mild higher inflammation for the hydrophobic salt. Whole-blood analysis highlighted an unreported high partition of amikacin in blood components up to 48 h, while significant lung levels were measured up to 72 h. Such a new observation was considered responsible for the nearly overlapping pharmacokinetic profiles of the two treatments. To overcome such an issue, a dry powder in an inhalable form may be best suited. Moreover, if confirmed in humans, and considering the current once-a-day regimen for amikacin aerosols, important yet-to-be-explored clinical implications may be postulated for such amikacin persistence in the organism.

## 1. Introduction

Current emerging multidrug resistance and the related antibiotic crisis demand immediate action [1]. To contrast difficult-to-treat lung infections, new effective therapeutic strategies need to be sought. In this scenario, direct antibiotic delivery at the site of infection through inhalation is gaining credit, owing to enhanced drug accumulation in the lungs and a parallel reduced chance of systemic side effects [2,3]. However, unfavorable drug physicochemical properties may hamper proper retention and penetration of the inhaled antibiotic in the respiratory tract, in turn increasing the risk of sublethal drug concentrations and the emergence of resistant bacterial strains. In this regard, enhancing drug hydrophobicity has been proposed as an advantageous strategy to increase bacteria killing while overcoming resistance by interfering with bacterial virulence factors [4,5,6].

Moreover, hydrophobic drugs have a higher chance to counteract infection niches by enhanced tissue and cell penetration [7,8].

Previous studies highlighted that the hydrophobic ionic coupling with oleic acid [9], metals [10,11,12] and deoxycholic acid (DCA) [13] of well-known hydrophilic antibiotics can increase bacteria-killing and antibiofilm action. Following this principle, we have synthesized a novel amikacin (Amk)-DCA hydrophobic salt with good activity and stability.

Amk can treat a wide range of infections by binding the ribosomal 30 s subunit and inhibiting protein synthesis [14]. The literature on Amk pulmonary delivery includes mainly liquid aerosols [15,16,17,18] and liposomes [19,20,21,22,23,24] and sporadic works on solid lipid nanoparticles [25] and self-emulsifying drug delivery systems [26]. An inhalable liposomal form of Amk (Arikayce^®^) has found recent approval by the FDA for pneumonia treatment [20,21,27]. Based on such premises, the present work aimed at exploring the in vivo behavior of Amk-DCA by investigating its pharmacokinetics (PK) and effects upon repeated intranasal (i.n.) administration by determining for the first time Amk in whole blood. It is worth noting that all the methods published so far for Amk quantitation have been performed only in plasma samples, thereby limiting a complete picture of Amk partitioning in blood. Indeed, drug dosage in whole blood is commonly considered rather difficult for routine analyses; for this reason, plasma has been and is still the mainstay surrogate for PK measurements. However, we here demonstrate that truthful PK data on this drug (irrespective of the administered form) cannot be accessed by using plasma as a simplified matrix. In this connection, in order to fill this gap, we have performed, for the first time, Amk analysis in whole blood for both chemical forms, trying to lay the initial basis for a more accurate and comprehensive evaluation of this old drug under a new delivery perspective.

## 2. Materials and Methods

### 2.1. Materials

Amikacin disulfate was purchased from Alfa Aesar (Milan, Italy), and sodium deoxycholate, glycine, tris(hydroxymethil)aminomethane (TRIS) and 1-fluoro-2,4-dinitrobenzene (FDNB) were obtained from Merck Life Science (Milan, Italy). Water was purified by reverse osmosis. All organic solvents and the eluent components for the HPLC study were of analytical grade and purchased from Merck Life Science (Milan, Italy).

### 2.2. Preparation of the Amk-DCA Hydrophobic Salt

The Amk-DCA hydrophobic salt was prepared according to the method detailed by Giovagnoli et al. [13]. In order to ensure administration reproducibility during the in vivo experiments, the Amk-DCA salt was further micronized using a PilotMill-Zero micronizer (Food Pharma System, Como, Italy) at 3.0 bar pressure for up to 2 h.

### 2.3. Animals

Female healthy CD1 mice aged 6–7 weeks and weighing 23 ± 2 g (Charles River Laboratories, Wilmington, MA, USA) were housed with a 12 h light/12 h dark cycle. Room temperature was maintained at 21 ± 1 °C and relative humidity between 40% and 60%. Mice had *ad libitum* access to food and water. This study was approved by the Academic Ethical Committee and the Italian Ministry of Health (authorization number: 432/2018-PR).

### 2.4. Pharmacokinetic Study

A single-dose PK study was performed in healthy mice after i.n. instillation of free Amk and Amk-DCA at 8 mg/kg dose [28]. Sixty-three mice were randomly divided into 3 groups: (1) a control group treated with physiological solution (*n* = 3), (2) a group treated with Amk solution (*n* = 30), (3) a group treated with Amk-DCA suspension (*n* = 30). Appropriate amounts of Amk and Amk-DCA salt were dissolved and suspended, respectively, in 1.0 mL of physiological solution prior to administration. Each mouse received i.n., by means of a micropipette, 20 μL of physiological solution, Amk-solution or Amk-DCA suspension. After subcutaneous anaesthetization, six animals were sacrificed at predetermined time points (0.5, 2, 6, 24 and 48 h after treatment). Blood was obtained by cardiac puncture and collected into EDTA-containing tubes, and lungs were surgically removed and collected in 1.5 mL Eppendorf tubes. All samples were stored at −80 °C until use.

A model-independent analysis was employed to calculate pharmacokinetic parameters. The maximum drug concentrations (C_max_) and the associated time points (T_max_) were determined by direct observation of the drug concentration vs time curve. The 0–48 h area under the curve (AUC_0–48h_) values were calculated using the trapezoidal rule (Prism Version 8, GraphPad). Data were expressed as mean ± S.D (*n* = 6).

### 2.5. Repeated Administration Regimen

Two repeated doses of 2 mg/kg free Amk and Amk-DCA were administered to healthy mice by i.n. instillation. Twenty-nine mice were randomly divided into three groups, as reported for the PK study: (1) a control group treated with physiological solution (*n* = 10), (2) a group treated with Amk solution (*n* = 9), (3) a group treated with Amk-DCA suspension (*n* = 10). All mice were i.n. treated as above with two consecutive administrations 48 h apart. Mice were sacrificed, after subcutaneous anaesthetization, 24 h after the second administration. Blood and tissue samples were collected and stored as reported above.

### 2.6. Inflammatory Cytokines and Histology

Lung samples were collected, homogenized and centrifuged at 11,000 rpm for 10 min at 25 °C with the aim to remove cellular fractions. Supernatants were then tested for interleukin-1β (IL-1β) and interleukin-6 (IL-6) levels by specific ELISA kits following the manufacturer’s instructions (eBioscience, Thermo Fisher Scientific, San Diego, CA, USA). For histology, the lungs were excised and immediately fixed in 10% neutral buffered formalin. The fixed organs were dehydrated, embedded in paraffin and sectioned longitudinally. Then, 3–5 µm-thick sections were mounted on glass slides and stained with hematoxylin/eosin to visualize histological signs of inflammation by Leica DM2500 model light microscopy (Leica Microsystems, Wetzlar, Germany).

### 2.7. Sample Preparation and Analysis

Blood samples were submitted to sonication into an ice bath for 5 min to allow complete cell lysis. Blood was deproteinized with an appropriate volume of 5% *v*/*v* ammonium hydroxide solution in acetonitrile and centrifuged at 15,000 rpm for 25 min at 5 °C. The supernatant was collected, dried up under vacuum at 40 °C and then resuspended in a proper volume of bidistilled water. Lung lobes were ground with a Potter pestle until obtaining a mush, deproteinized with a volume of 0.6 M perchloric acid (3:1 *v*/*w*_lung tissue_) and centrifuged at 15,000 rpm for 25 min at 5 °C. The supernatant was collected, dried up under vacuum at 40 °C and resuspended in a proper volume of bidistilled water. To check for drug partitioning, fresh blood samples were immediately centrifuged at 15,000 rpm for 10 min at a temperature of 5 °C. Plasma and the pellets were frozen and stored at −80 °C until analysis.

Samples were analyzed by high-performance liquid chromatography (HPLC) using a published validated method with only minor modifications [29]. The stationary phase was a Robusta C18 (250 × 4.6 mm, 5 μm, from SepaChrom, Rho, Italy) set up at 45 °C. The mobile phase, composed of acetonitrile/water/acetic acid 47:53:0.1 *v/v/v*, was flowed at a flow rate of 1.0 mL/min for the quantification of Amk in whole-blood and plasma samples, whereas in lung tissue, a gradient elution mode was set up (Eluent A: acetonitrile/water/acetic acid 47:53:0.1 *v/v/v*; Eluent B: 0.1 v acetic acid in water). Before starting with the gradient program, the column was equilibrated with 20% A. The final gradient program, at a flow rate of 1.0 mL/min, was the following: 0–8 min linear gradient from 20% to 100% A; 8–30 min, 100% A; 30–31 min, linear gradient to 20% A; 31–45 min, 20% A (column re-equilibration step to the starting conditions). The drug was detected using a photodiode-array (PDA) detector at 365 nm after precolumn derivatization with FDNB in the presence of TRIS solution (1% *w/v*) and dimethyl sulfoxide described in detail by Papp et al. [30].

### 2.8. Statistical Analysis

All data were analyzed by Student’s *t*-test using Prism (Version 8, GraphPad). The significance level was set at 0.05. The deviations of the computed AUCs were calculated based on the method reported by Bailer [31] and Gragnon [32].

## 3. Results and Discussion

**Figure 1:**
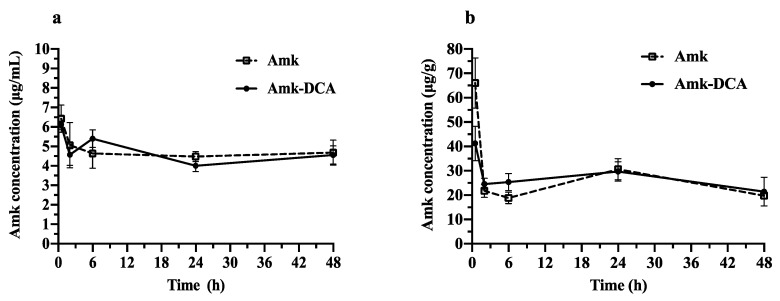
Drug concentration–time profiles after i.n. instillation of the Amk solution and the Amk-DCA suspension for (**a**) blood and (**b**) lung tissue (mean ± standard deviation) (*n* = 6). The dose of 8 mg/kg was chosen based on human clinical studies [33].

The blood and lung tissue concentration of the Amk solution and Amk-DCA suspension vs. time profiles, as well as the PK data following i.n. administration, are illustrated in Figure 1 and Table 1, respectively. For both Amk and Amk-DCA, rapid absorption into the systemic circulation was observed, with a C_max_ within the first 30 min. The lung C_max_ was significantly higher for the Amk solution compared to the Amk-DCA suspension (66.0 ± 10.4 µg/g_lung tissue_ and 41.3 ± µg/g_lung tissue_, respectively, *p* = 0.0011). However, Amk-DCA was slightly more retained in the lung, with an increased residence time between 6 and 24 h (*p* = 0.0050). Similar AUC_0-48h_ values of 1204.0 ± 76.2 and 1275.0 ± 126.8 µg h/g were obtained for Amk and Amk-DCA, respectively. As expected, the lung tissue/blood AUC_0–48h_ ratios of 5.6 and 5.9 for Amk and Amk-DCA, respectively, clearly indicated higher accumulation in the lungs, with an unexpected nonsignificant difference between the free drug and Amk-DCA. It is worth noting that drug levels, either in blood or in the lung tissue, remained constant throughout the 48 h time period investigated, making the estimation of the extrapolated AUC (AUC_0–∞_), half-life (t_1/2_), and elimination rate constant not possible.

As a limitation of this study, with 30 min being the first time point and corresponding to T_max_, an earlier C_max_ cannot be ruled out. However, possible differences in T_max_ in this narrow time frame are not likely to be clinically relevant.

The comparable profiles for Amk and Amk-DCA, either in the lungs or blood, may suggest that Amk-DCA delivered as a suspension dissociates in the biological fluids, allowing the drug to follow its natural biological fate. However, with this hypothesis being in contrast with the Amk-DCA in vitro stability observed at concentrations lower than the minimum inhibitory concentration (MIC_90_) measured against *Staphylococcus aureus* strains [13], further studies are planned to evaluate such an instance.

As a second part of the study, two repeated i.n. administrations were carried out using a four-fold-lower dose (2 mg/kg) in place of the 8 mg/kg dose initially selected for the PK evaluation. Indeed, the higher dose is unsuited for multiple administrations as it is a clinical dose delivered by an aerosol over an extended period of time, whereas in this study, amikacin was delivered by a one-shot i.n. administration.

**Figure 2:**
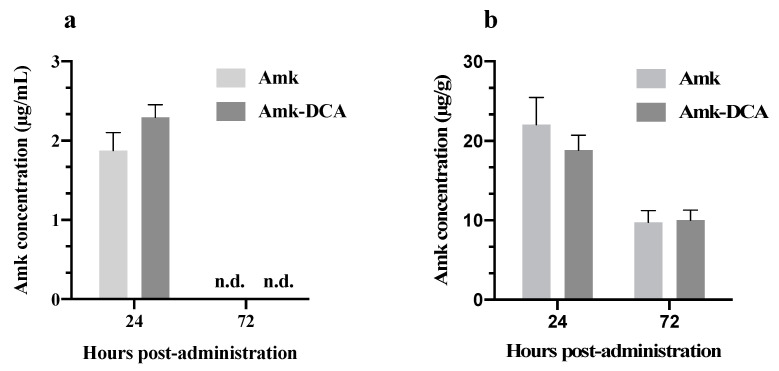
Drug levels (**a**) in blood and (**b**) lung tissue, measured at 24 h and 72 h after two repeated i.n. administrations at 2 mg/kg dose (mean ± standard deviation, *n* = 2), n.d. not detected at the retention time of standard.

Interestingly, in line with the above-reported prolonged retention either in blood or lung tissue (Figure 1), blood and lung levels after two consecutive administrations showed the tendency of the drug to accumulate in both compartments, with a prevalence in the lung tissue. Indeed, not consistently with the Amk plasma t_1/2_ in mice of less than 30 min [34], drug concentrations, measured 24 h after the second administration, ranged between 1.8 and 2.3 μg/mL in blood and 23 and 18 μg/g in the lungs (Figure 2a,b). The amounts are nearly two and three times higher, respectively, than those expected, based on the levels obtained at 24 h in the PK study. Extending the analysis to 72 h after treatment, Amk was undetected in blood, and it was still well measurable in the lung tissue, with levels close to 10 μg/g (Figure 2b). This preferential retention in the lungs was expected due to the modality of administration. However, the behavior was not significantly different between the two treatments, supporting the hypothesis that, in both cases, the drug in the free form binds to cells and tissues, which is coherent with the above-theorized dissociation of Amk-DCA in physiologic fluids.

**Figure 3:**
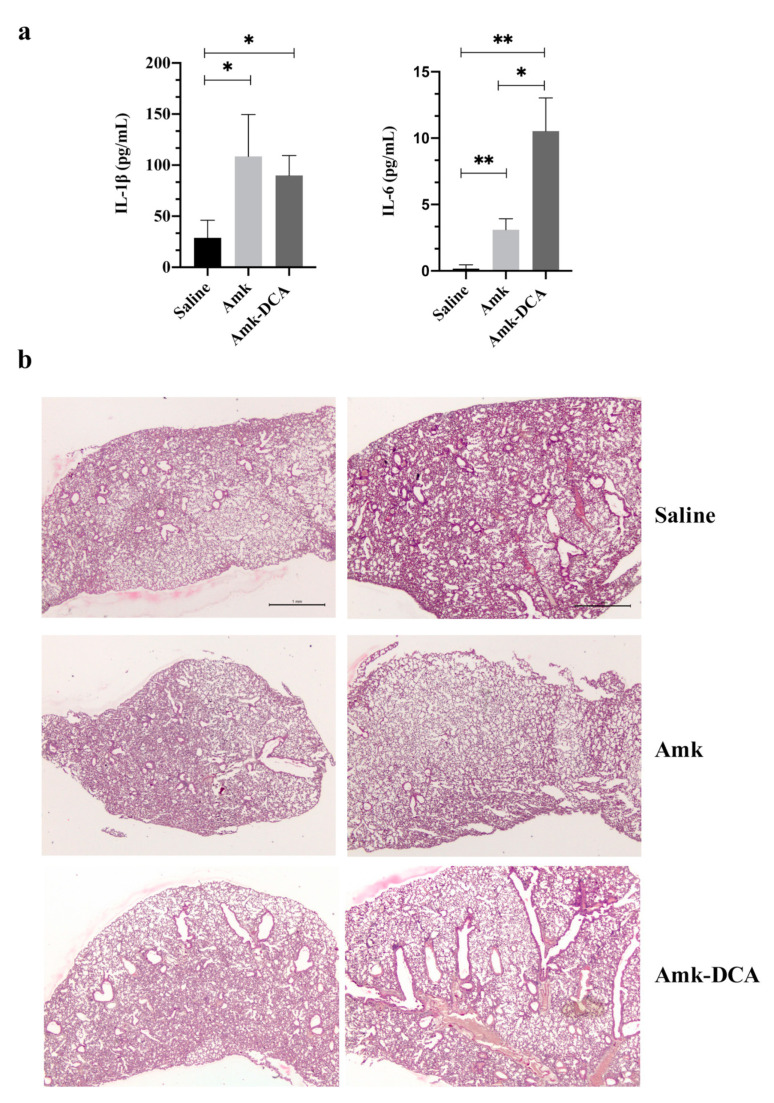
(**a**) Levels of proinflammatory cytokines IL-1β and IL-6 in the lung homogenates after two repeated i.n. administrations of the vehicle (saline), Amk solution and Amk-DCA suspension at 2 mg/kg dose (mean ± standard deviation, *n* = 3). (**b**) Histological assessment of mouse lung tissue after i.n. instillation with sterile saline (control mice), Amk and Amk-DCA (100× magnification), * *p* ≤ 0.05, ** *p* ≤ 0.01.

The effect of the novel Amk-DCA salt on the airway inflammatory response after two repeated administrations was assessed by measuring proinflammatory IL-1β and IL-6 cytokines in lung homogenates (Figure 3a). For both treatments, the expression of the two cytokines increased compared to the control. However, only IL-6 was significantly higher in Amk-DCA-treated mice compared to Amk, and the IL-1β level was comparable for both treatments. The increase in IL-6 and not IL-1β expression may suggest a mild proinflammatory effect of Amk-DCA compared to the free drug. In fact, while high IL-1β levels generally correlate with proinflammatory events and tissue damage [35], higher IL-6 expressions have been associated even with protective effects against direct lung injury [36,37]. Studies have been already planned to deepen the knowledge on this controversial aspect.

Further supporting a low inflammatory effect, histological microphotographs showed the normal architecture of the lung tissue in both treatments, albeit aggregation areas of inflammatory infiltration in mice treated with Amk and signs of hyperplasia in those treated with Amk-DCA were observed, with no major differences between treatments (Figure 3b).

Overall, these results seem to suggest that Amk-DCA was well tolerated upon challenging the mice with two repeated administrations via the pulmonary route. This observation is important considering the variable toxicity of bile acids that remains a major concern, regardless of their reported potentiality in pulmonary drug delivery [38,39,40,41].

Finally, in order to investigate the reason behind the observed high and extended Amk retention observed in blood, the above-mentioned method for drug quantitation in whole blood was used to analyze plasma and blood cell pellets 24 h after treatment.

**Figure 4:**
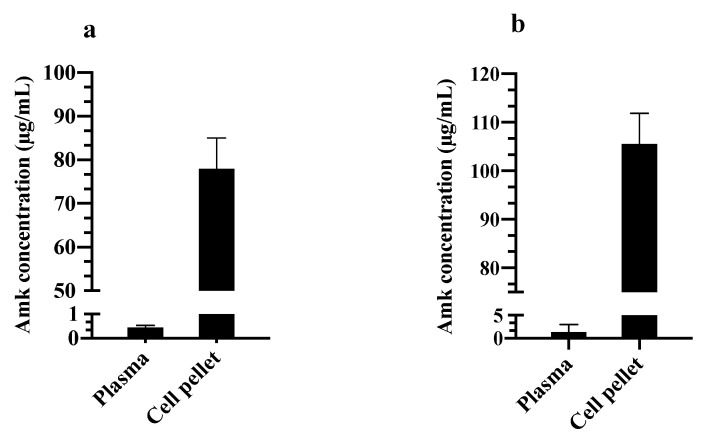
Drug levels in plasma and blood cell pellets measured at 24 h after i.n. administration of the Amk solution (**a**) and the Amk-DCA suspension (**b**) (mean ± standard deviation, *n* = 3).

The results showed high drug partitioning in favor of blood cells for both Amk and Amk-DCA (Figure 4a,b). Such an observation correlated with a blood plateau level/C_max_ ratio of about 60%, calculated from the PK study, which suggests high drug retention in blood components. To the best of our knowledge, Amk partitioning in the blood cell compartment (Figure 4a,b) has never been reported so far, and this phenomenon may explain the observed Amk residence up to 48 h in blood and 72 h in lung tissue. Therefore, this observation is new and may account for the almost overlapping PK profiles of the two treatments. In fact, Amk binding seems to be independent of the administration form (solution or suspension, free drug or salt), once more suggesting that Amk-DCA may dissociate in physiologic fluids when administered i.n. as a suspension.

Considering the long history of aminoglycosides’ clinical use, this lack of knowledge is somewhat surprising since accumulation phenomena have already been described in human tissues upon the systemic administration of gentamicin [42,43] and amikacin as well in animal models [44,45]. Hypotheses include the binding to cells through a Schiff’s base bond formation [46]. This knowledge gap unavoidably stems from the common use to perform analyses only on plasma samples or serum, which has led to overlooking such a potentially critical phenomenon.

Even though the clinical relevance of this finding is yet to be unraveled, and further studies are required, based on previous reports, similar behavior cannot be excluded in humans as well. This possible scenario warns for a critical assessment of the Amk treatment regimen when either administered systemically or inhaled. In fact, considering the current once-a-day regimen for Amk aerosols, adjustments in dosing frequency may be required to prevent adverse events associated with drug retention and progressive accumulation in tissues and blood. Moreover, to counteract the important tissue-binding effect, the administration of Amk-DCA as an inhalable dry powder could be worthwhile.

## 4. Conclusions

This work, somehow unexpectedly, delivered novel information on Amk binding to blood components while confirming the high drug retention in lung tissues. In fact, Amk quantitation, performed for the first time in whole blood, enabled the observation of considerable blood cell binding that has never been reported before, despite potential high clinical relevance.

Such a high drug partitioning was considered responsible for the nearly overlapping PK profiles of free drug and Amk-DCA, which may suggest dissociation over time of the salt in physiologic fluids. Being seemingly tolerated upon pulmonary administration, Amk-DCA dry powder formulation may be preferred to improve PK and influence drug binding and partitioning. Such a possibility is currently under evaluation in our lab.

If such binding is confirmed in humans, based on previous long-dated literature warning for potential aminoglycoside accumulation in humans [42,43,46], and considering the current once-a-day inhaled Amk treatments [28], rethinking the current regimen may be recommended to prevent important side effects over prolonged exposure. Further studies are planned to untwine this noteworthy as well as critical aspect.

## Figures and Tables

**Table 1:** Lung and blood pharmacokinetic parameters after i.n. instillation of Amk and Amk-DCA in CD1 mice (mean ± S.D., *n* = 6).

Parameter	Amk Solution		Amk-DCA Suspension	
	Lung	Blood	Lung	Blood
C_max_ (μg/mL or μg/g)	66.0 ± 10.4	6.4 ± 1.7	41.3 ± 7.1	6.1 ± 0.5
T_max_ (h)	0.5	0.5	0.5	0.5
AUC _0–48h_ (μg h/mL or μg h/g)	1204.0 ± 76.2	219.6 ± 44.1	1275.0 ± 126.8	217.5 ± 22.3
AUC _lung_ to AUC _blood_ ratio	5.6 ± 1.1		5.9 ± 1.1	

## Data Availability

The data presented in this study are available on request from the corresponding author. The data are not publicly available due to institutional policy.
